# *Toxoplasma gondii* and *Trypanosoma lewisi* Infection in Urban Small Mammals From Cotonou, Benin, With Special Emphasis on Coinfection Patterns

**DOI:** 10.1155/tbed/9976509

**Published:** 2025-02-14

**Authors:** Jonas R. Etougbétché, Gualbert Houéménou, Antoine A. Missihoun, Philippe Gauthier, Henri-Joël Dossou, Lokman Galal, Ambroise Dalecky, Christophe Diagne, Gauthier Dobigny, Aurélien Mercier

**Affiliations:** ^1^Laboratory of Applied Biology Research, Polytechnic School of Abomey-Calavi, University of Abomey-Calavi, Cotonou, Benin; ^2^Laboratory of Molecular Genetics and Analysis of Genomes, Faculty of Sciences and Techniques, University of Abomey-Calavi, Cotonou, Benin; ^3^CBGP, IRD, CIRAD, INRAE, Institut Agro, University of Montpellier, Montpellier, France; ^4^Living Environment Institute (ICaV), Université of Abomey-Calavi, Cotonou, Benin; ^5^U1094 Inserm, U270 IRD, EpiMaCT—Epidemiology of Chronic Diseases in Tropical Zone, Institute of Epidemiology and Tropical Neurology, CHU Limoges, Omega Health, University of Limoges, Limoges, France; ^6^IRD, Aix Marseille University, LPED, Marseille, France; ^7^BIOPASS 2, IRD, UGB, Cirad, ISRA, UFR S2ATA, Saint-Louis, Senegal; ^8^Plague Unit, Institut Pasteur of Madagascar, Antananarivo, Madagascar

**Keywords:** health ecology, multihost multiparasite communities, small mammals, *Toxoplasma gondii*, *Trypanosoma lewisi*, urban eco-epidemiology, zoonotic infectious diseases

## Abstract

A growing number of studies has highlighted the importance of coinfections in eco-evolutionary processes underlying host–parasite interactions and the resulting epidemiology of zoonotic agents. Small mammals, and particularly rodents, are known to be important reservoirs of many zoonotic pathogens, such as *Toxoplasma gondii* and *Trypanosoma lewisi*, that are responsible for toxoplasmosis and atypical trypanosomiasis in humans, respectively. Laboratory experiments on rodent models have shown that primary infection with *T. lewisi* increases the host sensitivity to other parasites, including *T. gondii*, following an alteration in the immune response. However, data on potential interactions between these parasites in wild small mammals remain scarce. In this study, we determined the *T. lewisi* prevalence in 553 small mammals from four localities of Cotonou city, Benin. The results were then combined with *T. gondii* data previously collected for the same individuals in order to investigate the influence of *T. lewisi* on *T. gondii* infection, and vice versa, using co-occurrence tests and generalized linear mixed models (GLMMs). Despite quite high overall prevalence (32.5% and 15.2% for *T. lewisi* and *T. gondii*, respectively), we observed a clear and significant segregation between the two parasites. This may be explained by (i) differences in the species-specific receptivity and/or sensitivity of small mammal host species to infection by these two parasites, with *Rattus rattus* (Rra), *Rattus norvegicus* (Rno), and *Mastomys natalensis* (Mna) being the main hosts of *T. lewisi*, while *Crocidura olivieri* (Cro) and *Mus musculus domesticus* (Mus) were the main hosts for *T. gondii*; and/or (ii) a possibly high mortality in coinfected animals in the wild. Although dedicated experimental studies are required to confirm this pattern, as they stand, our data fail to support that in nature, the infection of small mammals by one of these two parasites favors widespread infection by the second one.

## 1. Introduction

Within-host interactions between parasites may strongly influence pathobiome dynamics and play a major role in structuring both parasite and host populations [1, 2]. Such interactions can have important repercussions on the ecology of zoonotic pathogens, hence, on human health, for example, by altering the host reservoir susceptibility (including receptivity and sensitivity), modifying the temporal dynamics of infections, increasing transmission risks, or impacting the pathogen virulence [1]. Multiparasitism is common in all animal organisms, and rodents have been particularly used as model hosts for studies on infection by multiple pathogens [3–7], especially in domestic areas where they are key reservoirs for a wide panel of zoonotic pathogens [3, 4, 7, 8].

*Toxoplasma gondii* and *Trypanosoma lewisi* are two protozoan parasites of worldwide distribution which are responsible for toxoplasmosis [9] and atypical trypanosomiasis in humans [10, 11], respectively. Human infection with *T. gondii* usually occurs through consumption of oocyst-contaminated vegetables or undercooked meat, while primary infection during pregnancy may result in congenital transmission ([12]; reviewed in [13, 14]). Toxoplasmosis is usually asymptomatic [15–17] and up to one-third of humans may be infected globally [18–20].

However, clinical forms are sometimes observed, especially in immuno-compromized patients and fetuses [21–24] as well as in immuno-competent individuals from tropical regions infected with *T. gondii* atypical strains that circulate specifically in the environment of these geographical areas [25–27]. *Trypanosoma lewisi* is transmitted through the feces of infected ectoparasitic fleas that acts as vectors of parasite dissemination among mammals, especially rodents. A few pathogenic and even lethal cases of human infection have been described in Asia and Africa; but the global impact of *T. lewisi* on human health may be widely underestimated and remains to be fully documented [10, 11, 28, 29]. In addition to virulence factors of *T. gondii* infecting strain and host-specific genetic factors, coinfections involving *T. gondii* and other parasites are also likely to influence the ecology of toxoplasmosis and virulence phenotype [30–34]. If true in the wild, this would have important consequences for parasite ecology.

Although they display very different transmission modes, previous studies have shown that both parasites can circulate within common environments, especially in tropical areas where they can share the same reservoir hosts, especially rodents [7, 35]. Thus, *T. gondii* has been identified in several commensal rodent species [36], including those investigated recently in Cotonou, Benin [37]. Rodents are also the main reservoirs of *T. lewisi* in Africa, especially the invasive genus *Rattus*, which has been proposed to play a special role in its ecology and dissemination across the continent [38–41]. Expectedly, it was also detected in many rodents from Cotonou [42] since rats were particularly abundant [43] and the parasite has already been observed in rats from similar socioenvironmental contexts in Niger, Nigeria, and Senegal [41, 44]. Both parasites were also found in African shrews of the genus *Crocidura*, in Cotonou city [37, 42]. Keeping in mind their usually quite high prevalence in small mammals: for example, 15.2% for *T. gondii* [37] and 57.2% for *T. lewisi* [42], the concomitant presence of both parasites in Cotonou city may provide valuable models for further investigation of the role of coinfections in eco-evolutionary fate of zoonotic pathosystems in urban reservoir hosts communities.

In this study, we took advantage of an already existing small mammal-borne *Toxoplasma* dataset from Cotonou [37], to assess *T. lewisi* presence/absence in the same rodent individuals, and then to specifically investigate the relationships between *Toxoplasma* and *Trypanosoma* infections in urban wild small mammals taking into account a panel of biological and environmental potentially confounding factors.

## 2. Materials and Methods

### 2.1. Data Collection

During 2017 and 2018, a study was conducted on the circulation of *T. gondii* in small mammals from four localities of Cotonou [37]. We here took advantage of these already available small mammal samples to investigate the coinfection patterns between *T. lewisi* and *T. gondii*. Sampling sites and trapping procedures were previously described in detail [37]. In brief, field campaigns were conducted in three socioenvironmentally contrasted inner-city districts, namely, Ladji, Agla, and Saint-Jean (in October 2017 and June 2018) on the one hand, and in Cotonou seaport (Autonomous Seaport of Cotonou (ASC)) area (in September–November 2017 and March 2018) on the other hand. In each of the three districts, 9–11 households (hereafter, designated as “district sites”) were investigated (see details in [42, 45]), while nine observatory sites were sampled in ASC (hereafter, designated as “ASC sites”; see [46] for their complete description). Each individual was unambiguously identified at the species level using morphological, DNA sequencing, and/or microsatellite genotyping (see details in [37, 42, 45, 46]). Sex and age (adult vs. juvenile) were assessed following criteria associated with the body mass and signs of sexual maturity as described elsewhere [37, 47]. The presence and number of ectoparasitic fleas was assessed following fur brushing as described elsewhere [48, 49].

We also took advantage of a previous study relying on the same experimental design to obtain socioenvironmental data including landcover data and social uses associated with buildings, as well as surface water occurrences (see details in [37, 50]). Here, we focused on the 553 individuals that had already been investigated for the presence of *T. gondii* using molecular detection (see detailed protocols in [37]) and *T. gondii* prevalence data (Table S1). Note that these 553 animals are all different from the 369 small mammals used in a previous study on small mammal-borne *T. lewisi* from Cotonou [42] for which no data on *Toxoplasma* were available.

### 2.2. Molecular Detection of *Trypanosoma lewisi*

Total genomic DNA was extracted from the spleen using the Qiagen Extraction Kit (DNeasy 96 Blood and Tissue Kit) according to the manufacturer's recommendations. DNA elution was performed in 200 µL of buffer AE. The presence of *T. lewisi* DNA was checked in independant duplicate through a qPCR protocol previously described [38, 41]. The latter procedure targets a 131 bp-long fragment of the *Trypanosoma* 18S rDNA gene fragment, using primers Trypano 1 (5′-AGGAATGAAGGAGGGTAGTTCG-3′) and Trypano 2 (5′-CACACTTTGGTTCTTGATTGAGG-3′) as well as two hybridization probes (Trypano 3: [LC640] AGAATTTCACCTCTGACGCCCCAGT [Phos] and Trypano 4: GCTGTAGTTCGTCTTGGTGCGGTCT [Flc]). Genomic DNA extracts from *T. lewisi* and *T. brucei* cell cultures were used as positive controls, while sterile water served as a negative control. The sigmoidal shape of each amplification curve was checked visually in order to discard nonsigmoidal signals that may represent false positive results. All individuals that provided at least one positive signal (out of the two duplicate qPCR experiments) were considered *Trypanosoma*-positive. The qPCR results were expressed as cycle threshold values. *Trypanosoma*-positive samples with sufficient DNA (Ct ≤ 30) were genotyped relying on nine *T. lewisi*-specific microsatellite markers (LEW2, LEW12, LEW16, LEW32, LEW35, LEW42, LEW44, LEW53, and LEW55) recently developed [51]. For this purpose, PCR amplifications and sequencing was performed as described in Ségard and colleagues and results were analyzed using GeneMapper 4.1 software packages (Applied Biosystems). The genotypes obtained were compared with the Ref-Wery reference genotype for unambiguous *T. lewisi* molecular identification.

### 2.3. Data Analysis

Chi-square tests were used to compare parasite prevalence between host species and/or between trapping localities. We carried out these tests for *T. lewisi* prevalence (between host species and between localities) on the one hand, and for *T*. *gondii*–*T. lewisi* coinfection (between and within host species) on the other hand. We then performed two complementary sets of analyses to explore the possible interactions between both parasites in relation to host-intrinsic and host-extrinsic factors: co-occurrence analyses and generalized linear mixed models (GLMMs).

#### 2.3.1. Co-Occurrence Analyses

Co-occurrence analyses are used to test whether two entities are found statistically aggregated or statistically segregated more often than expected under random association [52, 53]. Here, such deterministic versus random associations of the two parasite species were tested depending on the host species or on the locality, as well as on the whole dataset combining all host species and localities. To do so, data were organized in several matrices following the different small mammal species and different sampled localities: each column corresponded to a host individual, while each row indicated the absence (0) or the presence (1) of a given parasite species (i.e., one row for *T. lewisi*, another one for *Toxoplasma*). Only matrices with at least 10 host individuals were considered in this analysis. Observed data were compared to expected results under the null hypothesis of random assembly with a 95% confidence limit [52, 54] in PAIRS v.1.0 [55] and using the standardized *Z*-score (ZCS) [56] as a quantitative index of co-occurrence. Significant negative and positive ZCS indicated aggregation and segregation, respectively [52]. Statistical significance was assessed by comparing the observed ZCS to values obtained from 10,000 iterations using a statistically recommended null model using the fixed row and equiprobable column constraints algorithm [52].

#### 2.3.2. GLMMs

Generalized linear mixed predictive models (GLMMs) were tested not only on the whole small mammal community, but also for each species with at least 50 individuals sampled, in order to explore the relationships between the prevalence of *T. gondii* and that of *T. lewisi* in small mammals across Cotonou city, taking geographic/environmental parameters into account. These analyses were performed separately for the three urban districts (herefater, designed as to “district-centered models”) on the one hand, and for the ASC (“ASC-centered models”) on the other hand, since (i) these two areas display very distinct socioeconomic, historical, and environmental characteristics, (ii) no landscape/GIS data was available for the ASC, and (iii) the trapping campaigns were not carried out exactly at the same period in the seaport and the inner city (see above). For each dataset, three models were tested with (1) the prevalence of *T. gondii*, (2) the prevalence of *T. lewisi*, and (3) the prevalence of coinfections as binary response variables, respectively.

In each model, we considered the individual characteristics of the host (sex, age, and presence/absence of fleas), the period of capture (i.e., trapping session), and socioenvironmental proxies (i.e., trapping sites coordinates along the first four principal components retrieved from the set of 21 GIS–based landscape metrics and available only for district-centered models) as explanatory variables. For the first two models, when the prevalence of *T. lewisi* was used as response variable, that of *T. gondii* was added to explanatory variables, and vice versa. Districts (in district-centered models) and ASC sites (in ASC-centered models) were considered as random variables in order to account for possible spatial variation or autocorrelation.

Models with all possible combinations of the terms included in the starting model were generated, and the most parsimonious model (i.e., the one explaining the highest part of the total variance with the fewest explanatory variables) was chosen among those selected within two AIC units of the best model retrieved [57]. The significance of explanatory variables and their interactions was determined by deletion testing and log-likelihood ratio tests and, when needed, by pairwise Wilcoxon rank sum tests with 95% family-wise confidence level. The final model was validated by the over-dispersion test, the graphical checking of normality and independence as well as variance homogeneity of residuals. These analyses were performed in R [58] using dedicated packages, namely, lme4 for GLMMs [59] and MuMIN for model selection [60].

## 3. Results

### 3.1. Sampling

**Figure 1:**
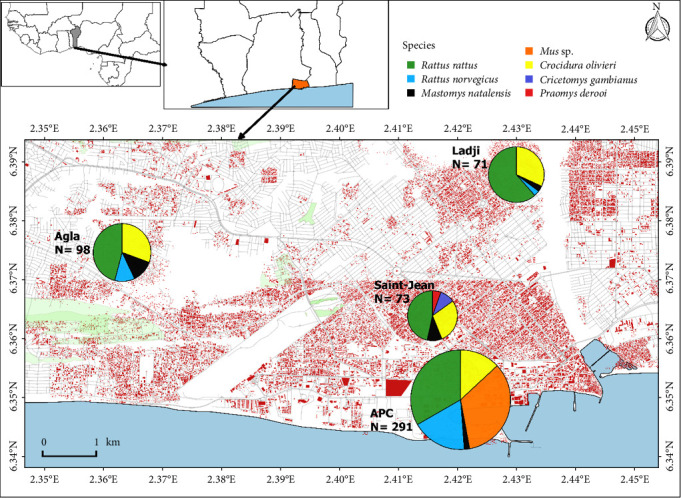
Species-specific distributions and relative abundances of the samples available for the present study (i.e., that were investigated for both parasites). Modified from Etougbétché et al. [37].

The two parasites *T. gondii* and *T. lewisi* could be concomittantly investigated in 553 individuals: 232 black rats (*Rattus rattus* (Rra)), 118 African giant shrews (*Crocidura olivieri* (Cro), 99 house mice (*Mus musculus domesticus* (Mus)), 66 brown rats (*Rattus norvegicus* (Rno)), 27 multimammate rats (*Mastomys natalensis* (Mna)), 7 Gambian pouched rats (*Cricetomys gambianus* (Cga)), and 4 Deroo's mice (*Praomys derooi* (Pde)). Among them, there were 406 adults and 115 juveniles (32 individuals displayed ambiguous patterns and their age could not be assessed with confidence), 245 males and 308 females. Eighty out of 553 (i.e., 14.5%) animals carried at least one flea with the highest prevalence of flea infestation observed in Rno (50%, i.e., 33 flea-carrying individuals out of 66). Captures per locality were distributed as follows: 98 small mammals in Agla (45 Rra, 30 Cro, 12 Mna, and 11 Rno), 91 in Ladji (56 Rra, 29 Cro, 3 Mna, and 3 Rno), 73 in Saint-Jean (34 Rra, 21 Cro, 7 Mna, 7 Cga, and 4 *P. derroi*), and 291 in ASC (99 Mus, 97 Rra, 52 Rno, 38 Cro, and 5 Mna; Figure 1 and Table 1).

### 3.2. Small Mammal-Borne *T. lewisi* Prevalence

Out of the 553 individuals screened, 181 were found *Trypanosoma*-positive, thus, representing an overall molecular prevalence of 32.7% (Table 1). One hundred and twenty-four (121) samples (Ct < 30) out of the 181 *Trypanosoma*-positive could be identified unambiguously as *T. lewisi* by microsatellite genotyping (data not shown).

The highest prevalence was found in Rra, Mna, and Rno with 55.2% (128/232), 44.4% (12/27), and 36.4% (24/66), respectively, with much lower prevalence in Mus (6/99; 6.1%) and Cro (9/118; 7.6%). A significant difference in *T. lewisi* infection was observed between host species (*χ*^2^ = 120.33, df = 4, *p* < 10^−3^) with black rats being more (*p* < 0.01) infected than other species, except Mna (*χ*^2^ = 0.73, df = 1, *p*=0.42). Cga and Pde showed only one and no individuals infected, respectively; however, they were very poorly represented in our dataset (*n* = 7 and *n* = 4) and were, thus, removed from subsequent analyses. Prevalences were significantly different between localities (*χ*^2^ = 8.5, df = 3, *p*=0.036), with small mammals from Ladji being the most infected ones (40/91; 44%), followed by those from St-Jean (27/73; 37%), Agla (32/98; 32.7%), and ASC (82/291; 28.2%).

### 3.3. Host Species-Specific Prevalence of *Toxoplasma gondii–Trypanosoma lewisi* Coinfection

Only 21 out of 553 (3.8%) of the studied individuals were found infected with both parasites (Table 1). Among these coinfected individuals, no significant differences (*χ*^2^ = 1.66, df = 1, *p*=0.19) were found between Rra (10/21; 47.7%) and Rno (5/21; 23.8%), but black rats were significantly more coinfected than the other species (*χ*^2^, all *p* < 0.04), while no difference was found between Rno and the other species where rather low prevalence were found: Cro (14.3%, i.e., 3/21) and Mna (9.5%, i.e., 2/21). Only one (1/99) Mus was found infected with both parasites. Comparisons of species-specific coinfection prevalence between localities have shown no significant difference in Rra (Fisher's exact, *p*=0.17) and in Cro (Fisher's exact, *p*=1). All coinfected Rno were found in ASC, thus, precluding any inter-locality investigation for this particular species.

### 3.4. Investigation of *Toxoplasma gondii–Trypanosoma lewisi* Coinfection Patterns

#### 3.4.1. Co-Occurrence Analysis

Most of the tests for co-occurrence of *T. gondii* and *T. lewisi* showed significant segregation between the two parasites, at both the host species and locality levels (all ZSCs >0 and all *p*-values ≤0.002, except for Mna that showed only marginally nonsignificant probability). Considering the whole small mammal dataset at the scale of Cotonou city the segregation pattern was also highly significant (Table 2).

#### 3.4.2. GLMM Analysis

Although several predictive models were tested (Table 3), in no instance did we find that the infection by one of the two parasites could be explained by the infection by the other one. This was true whatever the design of the model, the host species and the considered area.

#### 3.4.3. District-Centered Analysis

Considering all host species across the three urban districts, infection with either of the two parasite species could not explain the presence of the other. Examining coinfected species-specific patterns, only coinfection in Cro seemed to be related to the trapping session (*χ*^2^ = 6.04; *p*=0.014): they were significantly more commonly coinfected in October 2017 than in June 2018. Note that infection with any of the two parasites was significantly related to host species. Black rats were significantly less infected by *T. gondii* than other species (*χ*^2^ = 15.79; *p*=0.001; Wilcoxon test, *p* < 0.001). Infection with *T. lewisi* also varied between host species (*χ*^2^ = 41.29; *p* < 0.001): pairwise comparison of *T. lewisi* infection showed that Rra was significantly more infected than Cro and Rno, whereas Mna was more infected than Cro (Wilcoxon test, all *p*-values <0.01). No difference in infection between Rra and Mna was observed (*p*=0.75). In addition to host species, *T. lewisi* infection was also positively associated with the presence of ectoparasitic fleas (*χ*^2^ = 5.42; *p*=0.02) as well as partly with the landscape structure, namely, the first principal component (PC1) which constrasted “presence of dumpsites” with “houses” (*χ*^2^ = 5.6; *p* < 0.017). Focusing on the two best represented host species in our dataset, namely, Rra and Cro, we confirmed that only *T. lewisi* infection was significantly associated with the presence of fleas (*χ*^2^ = 4.05; *p*=0.04 and *χ*^2^ = 4.64; *p*=0.03, respectively).

#### 3.4.4. ASC-Centered Analysis

As for the urban districts, no statistically significant relationship between the two parasites was observed in ASC, whatever the host species considered. However, when considering all host species, the most parcimonious model best explaining coinfection included the age stage (*χ*^2^ = 5.7; *p*=0.016), with juveniles being more coinfected than adults. Furthermore, when all host species were considered, *T. lewisi* infection was also found significantly related to the host species in ASC (*χ*^2^ = 69.37; *p* < 0.001). The genus *Rattus* was once again found as the most infected one (Rra vs. Cro/Mus, Wilcoxon tests, both *p* < 0.001; Rno vs. Cro/Mus, Wilcoxon tests, both *p* < 0.001).

## 4. Discussion

Our study confirms the role of commensal small mammals in the large-scale circulation of two environnemental transmitted parasites within Cotonou City, namely, *T. lewisi* and *T. gondii* with overall molecular prevalences of 32.7% and 15.2%, respectively. Both parasites were observed in all investigated localities, although the level of their respective prevalence was variable from one to another. The implications of intrinsic and extrinsic factors on *T. gondii* infection have been extensively discussed in our previous study [37]. For this reason, here, we first discuss briefly some aspects of *T. lewisi* infection before tackling the socioenvironmental patterns that could explain concomitant presence of both parasites in some rodent and shrew individuals.

Our results are quite congruent with previous studies that already showed that *T. lewisi* was widespread among domestic and peri-domestic small mammals, with the black rat being the most widespread and important reservoir species in this part of West Africa [38, 41], including in Cotonou city [42]. As such, the overall qPCR–based prevalence observed within Cotonou by Dobigny et al. [42] and the present study were 57.2% (66.9% in black rats) and 32.7% (55.2% in black rats), respectively. However, contrary to Dobigny et al. study [42], we here found that Mna-specific prevalence (44.4%) was not statistically different from that observed in black rats. This observation seems to show that, in addition to the invasive genus *Rattus* usually considered as the main reservoir of *T. lewisi*, the native Mna may also play a major eco-epidemiological role in the maintenance and circulation of *T. lewisi* in urban environments. Species-specific prevalences observed in Cotonou appear higher than those retrieved from other African contexts using the same molecular detection technic: for example, 14.4% in Uganda (with 29.5% in black rats [40]); 14.6% in Niger and Nigeria (with 25.2% in black rats [41]), and 15.5% in Senegal (with 27.8% in black rats [44]). This not only indicates that conditions are right for rat to human transmission of *T. lewisi* in these socioenvironmentally degraded urban areas, but also that coinfection risk involving *T. lewisi* and other environmental or vector-borne infectious pathogens, would likely be heightened among domestic small mammals in Cotonou city.

In our study, we detected no significant association between landscape and small mammal-borne *T. lewisi*, thus, suggesting that this particular parasite may be widely distributed in most of the city. Considering the district-centered analysis, the GLMM performed on all species as well as those performed on the two most represented species, showed that *T. lewisi* infection was related not only to the host species (with rats as the main reservoirs), but also to the presence of fleas on the animals. These observations are not surprising since fleas are the main transmission vectors of *T. lewisi* in rodents in general, and particulary the genus *Rattus* [39, 61, 62].

Although both parasites were observed in Cotonou small mammal community (15.2% for *T. gondii* and 38.7% for *T. lewisi*), there was no sign of association, that is, favored coinfection. GLMM–based analysis showed no statistically significant relationship between both parasites species regardless of the strategies used. This supports the absence of coinfections that would be favored. By the contrary, a clear trend towards segregation was even observed by our co-occurrence analyses regardless of the host species (i.e., all or individual ones) and the locality considered. The segregation between the two parasites that we observed on the field could be explained with the species-specific composition of small mammals in the study sites, associated with differences in the host-species specific receptivity and/or sensitivity to these two parasites. Indeed, Rra appear less receptive to *T. gondii* infection than shrews and house mice, in which significantly higher prevalence levels were observed in Cotonou city [37]. Unfortunately, robust data on receptivity and sensitivity of these two parasites in African rodents are not available, thus, precluding any definitive conclusion.

The rarity of *T. gondii*–*T. lewisi* coinfected animals in our dataset (3.8%: 21 out of 553) could also be explained by a differential mortality of these individuals that would limit our ability to detect the concomitant presence of both parasites in the field. Indeed, if infection with *T. lewisi* leads to a severe alteration of the immune system, a second infection with *T. gondii* could be lethal, thus, drastically reducing the lifespan of coinfected animals. However, if this was to be true coinfection rates observed in natural small mammal populations would be greatly reduced. At the same time, several studies found that laboratory Norway rats infected with *T. lewisi* were more sensitive to infection by *T. gondii* [30–34]. Although this does not necessarily remain true for wild rodents, *a fortiori* for wild black rats, a *T. lewisi*-induced increased sensitivity to *T. gondii* would be expected to lead to an increase in the proportions of coinfected animals in absence of mortality [34]. Yet, this was not observed, thus, rather suggesting very high sensitivity leading to a very high mortality in coinfected animals or no coinfection. On the sole basis of our data, and in absence of experimental data on coinfection-associated mortality, it appears difficult to decide between the two explanations.

Indeed, several aspects may have weakened our study and the interpretation of its results. First, in murine models, experimental infections have demonstrated different levels of susceptibility/resistance to *T. gondii* between species or even lineages of the same species, which may depend on the host genetic background and/or the particular strain of *T. gondii* used for inoculations [63, 64]. Unfortunately, no such data are available for wild rodents and circulating *T. gondii* strains in Benin. Second, under natural conditions, knowing in which order did the parasites infect one given host is difficult, if not impossible. Yet, it is likely that a *T. gondii* infection preceding the one by *T. lewisi* will not induce the same immune response, hence, will not have the same physiological consequences than a *T. lewisi* infection followed by a *T. gondii* infection [34]. More generally, during their lifespan, wild rodents are very likely to be infected by several pathogens, some of them potentially strongly impacting their immune system and general condition, hence, the fate of subsequent encounters with other pathogens [1, 2, 65–70].

This suggests that study of coinfections in wild hosts should include the investigation of large panels of pathogens and parasites (e.g., through digestive tract analyses, highthrouput DNA sequencing approaches, etc.) when possible, taking into account the immune status of individuals since some parasite species could be eliminated by their hosts, knowing that antibody titers may decrease over time, becoming barely detectable or undetectable. In turn, this also implies the need for a very large number of sampled hosts in order to reach sufficient statistical power. Alternatively, experimental coinfections on wild or wild-derived rodents may allow one to investigate simplified coinfection processes and eco-evolutionary consequences in better controlled/documented frameworks.

Another limitation was that the qPCR method used to detect *T. lewisi* likely amplifies all *T. lewisi*-like species [38]. Microsatellite markers recently developed by Ségard et al. [51], which specifically identify *T. lewisi*, could only be applied to samples with Ct values <30. This limitation prevented the genotyping of 60 out of the 181 samples in our study. Nevertheless, for the 121 samples with Ct < 30, the technique developed by Ségard et al. [51] confirmed that all these samples were indeed *T. lewisi*. This finding corroborates results from previous studies, such as the analysis of 144 sequences from Cotonou, all of which were identified as *T. lewisi* [41, 42].

In conclusion, our study provides new insights into the interactions in natura between two urban small mammal-borne parasites with zoonotic potential in Africa, particularly in Benin. We confirmed the extensive circulation of *T. lewisi* among domestic small mammals within Cotonou city, especially in invasive genus *Rattus* and native Mna, thus, confirming potential spillover risk to city dwellers. We also observed a statistically significant segregation between *T. gondii* and *T. lewisi* in their hosts and an infrequent coinfection in Cotonou city, potentially due to differences in the receptivity of host species to infection by these two parasites and/or by a high mortality of coinfected individuals in the wild which would preclude their detection on the field. Experimental studies on wild rodent models are required to document further these two hypotheses. However, our results strongly suggest that, whatever the underlying process, infection by one of the two parasites is not a major driver of widescale and persistent infection by the second one in rodents.

## Figures and Tables

**Table 1:** Prevalence of *Toxoplasma gondii*, *Trypanosoma lewisi*, and coinfections by host species and sampled locality.

Localities	Parameters	All species	Rra	Rno	Mna	Mus	Cro	Cga	Pde
Agla	*N*	98	45	11	12	0	30	0	0
	Tox+ (%)	9 (9.2)	4 (8.9)	1 (9.1)	1 (8.3)	—	3 (10)	—	—
	Tryp+ (%)	32 (32.7)	23 (51.1)	0	6 (50)	—	3 (10)	—	—
	Tox+ &Tryp+ (%)	4 (4.1)	2 (4.4)	0	1(8.3)	—	1 (3.3)	—	—

Ladji	*N*	91	56	3	3	0	29	0	0
	Tox+ (%)	12 (13.1)	3 (5.4)	0	1 (33.3)	—	8 (27.6)	—	—
	Tryp+ (%)	40 (44)	32 (57.1)	2 (66.7)	2 (66.7)	—	4 (13.8)	—	—
	Tox+ &Tryp+ (%)	2 (2.2)	0	0	1 (33.3)	—	1 (3.4)	—	—

St-Jean	*N*	73	34	0	7	0	21	7	4
	Tox+ (%)	14 (19.2)	2 (5.9)	—	1 (14.3)	—	8 (38.1)	1 (14.3)	2 (50)
	Tryp+ (%)	27 (37)	21 (61.8)	—	3 (42.8)	—	1 (4.8)	2 (28.6)	0
	Tox+ &Tryp+ (%)	1 (1.4)	1 (2.9)	—	0	—	0	0	0

ASC	*N*	291	97	52	5	99	38	0	0
	Tox+ (%)	49 (16.8)	14 (14.4)	7 (13.5)	0	20 (20.2)	8 (21.1)	—	—
	Tryp+ (%)	82 (28.2)	52 (53.6)	22 (42.3)	1 (20)	6 (6.1)	1 (2.6)	—	—
	Tox+ &Tryp+ (%)	14 (4.8)	7 (7.2)	5 (9.6)	0	1 (1.0)	1 (2.6)	—	—

All localities	*N*	553	232	66	27	99	118	7	4
	Tox+ (%)	84 (15.2)	23 (9.9)	8 (12.1)	3 (11.1)	20 (20.2)	27 (22.9)	1 (14.3)	2 (50)
	Tryp+ (%)	181 (32.7)	128 (55.2)	24 (36.4)	12 (44.4)	6(6.1)	9 (7.6)	2 (28.6)	0
	Tox+ &Tryp+ (%)	21 (3.8)	10 (4.3)	5 (7.6)	2 (7.4)	1 (1.0)	3 (2.5)	0	0

*Note:* « Tox+ » and « Tryp+ » indicate the number of individuals infected only by *T. gondii* and *T. lewisi*, respectively, while « Tox+ &Tryp+ » correspond to coinfected ones.

Abbreviations: ASC, Autonomous Seaport of Cotonou; Cga, *Cricetomys gambianus*; Cro, *Crocidura olivieri*; Mna, *Mastomys natalensis*; Mus, *Mus musculus domesticus*; Pde, *Praomys derooi*; Rno, *Rattus norvegicus*; Rra, *Rattus rattus*.

**Table 2:** Co-occurrence of *Toxoplasma gondii* and *Trypanosoma lewisi* in small mammal species and sampled localities.

	Dataset	*N*	Try+	Tox+	Tox+&Tryp+	ZCS	*p*
Species	*Rattus rattus*	140	127	23	10	9.39	<0.001
	*Rattus norvegicus*	27	24	8	5	3.05	0.002
	*Mastomys natalensis*	13	12	3	2	1.83	0.067
	*Mus musculus domesticus*	24	20	5	1	4.73	<0.001
	*Crocidura olivieri*	33	27	9	3	5.12	<0.001

*Localities*	Agla	36	31	9	4	4.62	<0.001
	Ladji	50	40	12	2	7.53	<0.001
	St-Jean	40	27	14	1	8.13	<0.001
	ASC	116	81	49	14	10.96	<0.001

Total		242	179	84	21	16.45	<0.001

*Note: N*: number of individuals infected by at least one parasite; Try+ and Tox+: number of individuals infected only by *T. lewisi* and *T. gondii*, respectively; Tox+&Tryp+: number of individuals in which both parasites were detected.

Abbreviations: ASC, Autonomous Seaport of Cotonou; ZCS, standardized *Z*-score.

**Table 3:** Results of the best-fitted generalized linear mixed models (GLMMs) explaining *Toxoplasma gondii* and *Trypanosoma lewisi* mono-infections as well as coinfections in the different small mammal populations/species sampled.

Localities	Species^a^	Variable *r*éponse	Variables explicatives	*N* ^b^	Predictors^c^	Estimate ± SE	*χ* ^2^	*p*-Value
Districts (Agla, Ladji, and Saint-Jean)	All species	*Toxoplasma gondii*	*Trypanosoma lewisi*, species, sex, age, flea, session, PC1, PC2, PC3, PC4	246	Species	−1.78 ± 0.49	15.8	0.001
		*Trypanosoma lewisi*	*Toxoplasma gondii*, species, sex, age, flea, session, PCA1, PCA2, PCA3, PCA4		Species	2.2973 ± 0.4250	41.3	5.6e-09
					PCA1	0.2525 ± 0.1123	5.6	0.018
					Flea	1.5077 ± 0.6933	5.4	0.02
		Coinfection	Sex, age, flea, session, PCA1, PCA2, PCA3, PCA4		—	—	—	—
	*Rattus rattus*	*Toxoplasma gondii*	*Trypanosoma lewisi*, sex, age, flea, session, PC1, PC2, PC3, PC4	125	—	—	—	—
		*Trypanosoma lewisi*	*Toxoplasma gondii*, sex, age, flea, session, PC1, PC2, PC3, PC4		Flea	1.8149 ± 1.0865	4.05	0.04
		Coinfection	Sex, age, flea, session, PCA1, PCA2, PCA3, PCA4		—	—	—	—
	*Crocidura olivieri*	*Toxoplasma gondii*	*Trypanosoma lewisi*, sex, age, flea, session, PCA1, PCA2, PCA3, PCA4	78	—	—	—	—
		*Trypanosoma lewisi*	*Toxoplasma gondii*, sex, age, flea, session, PCA1, PCA2, PCA3, PCA4		Flea	35.70 ± 1.81e + 07	4.6	0.03
		Coinfection	Sex, age, flea, session, PCA1, PCA2, PCA3, PCA4		Session	34.58 ± 760.61	6.04	0.013

ASC	All species	*Toxoplasma gondii*	*Trypanosoma lewisi*, species, sex, age, flea, session	276	Session	1.0545 ± 0.3461	9.8	0.002
		*Trypanosoma lewisi*	*Toxoplasma gondii*, species, sex, age, flea, session		Species	—	69.37	5.808e-15
		Coinfection	Sex, age, flea, session		Age	1.5752 ± 0.6137	5.7	0.016
	*Rattus rattus*	*Toxoplasma gondii*	*Trypanosoma lewisi*, sex, age, flea, session	89	—	—	—	—
		*Trypanosoma lewisi*	*Toxoplasma gondii*, sex, age, flea, session		—	—	—	—
		Coinfection	Sex, age, flea, session		—	—	—	—
	*Rattus norvegicus*	*Toxoplasma gondii*	*Trypanosoma lewisi*, sex, age, flea, session	52	Age	2.9184 ± 0.9855	8.5	0.003
		*Trypanosoma lewisi*	*Toxoplasma gondii*, sex, age, flea, session		Age	2.2169 ± 1.0219	5.3	0.02
					Flea	−2.5288 ± 0.7233	15.1	0.0001
		Coinfection	Sex, age, flea, session		—	—	—	—
	*Mus musculus domesticus*	*Toxoplasma gondii*	*Trypanosoma lewisi*, sex, age, flea, session	95	Session	1.5474 ± 0.6678	6.7	0.009
		*Trypanosoma lewisi*	*Toxoplasma gondii*, sex, age, flea, session		—	—	—	—
		Coinfection	Sex, age, flea, session		—	—	—	—
	*Crocidura olivieri*	*Toxoplasma gondii*	*Trypanosoma lewisi*, sex, age, flea, session	35	—	—	—	—
		*Trypanosoma lewisi*	*Toxoplasma gondii*, sex, age, flea, session		—	—	—	—
		Coinfection	Sex, age, flea, session		—	—	—	—

*Note:* Flea: presence of fleas on the individual. Session: period of capture. PC 1–4: sites coordinates on landscape PCA axes 1–4 (see text and [50] for details).

Abbreviation: ASC, Autonomous Seaport of Cotonou.

^a^Host species considered in the model.

^b^Total number of individuals included in the model selection.

^c^Significantly selected variables.

## Data Availability

The data supporting the results of this study can be found in supporting information. Any other information or data could be provided upon request.

## Supporting Information

Supporting Information Table S1. Prevalence of T. *gondii*, T. *lewisi*, and coinfection by sex, age, session, and fleas porting. « Tox+ » and « Tryp+ » indicate the number of T. *gondii* and T. *lewisi* infected individuals, respectively, while « Tox+&Tryp+ » correspond to coinfected ones. “Rra,” “Rno,” “Mna,” “Mus,” “Cro,” “Cga,” and “Pde” stand for *Rattus rattus*, *Rattus norvegicus*, *Mastomys natalensis*, *Mus musculus domesticus*, *Crocidura olivieri*, *Cricetomys gambianus*, and *Praomys derooi*, respectively.
