# Supplementary material for: Toxoplasma gondii and Trypanosoma lewisi Infection in Urban Small Mammals From Cotonou, Benin, With Special Emphasis on Coinfection Patterns
Source: Transbound Emerg Dis. 2025 Feb 14;2025:9976509. doi: 10.1155/tbed/9976509 (PMC12016718; doi:10.1155/tbed/9976509)
Supplement: Supporting Information — Table S1. Prevalence of T. gondii, T. lewisi, and coinfection by sex, age, session, and fleas porting. « Tox+ » and « Tryp+ » indicate the number of T. gondii and T. lewisi infected individuals, respectively, while « Tox+&Tryp+ » correspond to coinfected ones. “Rra,” “Rno,” “Mna,” “Mus,” “Cro,” “Cga,” and “Pde” stand for Rattus rattus, Rattus norvegicus, Mastomys natalensis, Mus musculus domesticus, Crocidura olivieri, Cricetomys gambianus, and Praomys derooi, respectively. [file 9976509.f1.pdf]

Supplementary Table: Prevalence of *T. gondii*, *T. lewisi* and Co-infection by sex, age, session and fleas porting. « Tox+ » and « Tryp+ » indicate the number of *T. gondii*- and *T. lewisi* infected individuals, respectively, while « Tox+ &Tryp+ » correspond to co-infected ones. "Rra", "Rno", "Mna", "Mus", "Cro, "Cga" and "Pde" stand for *Rattus rattus, Rattus norvegicus, Mastomys natalensis, Mus musculus domesticus, Crocidura olivieri, Cricetomys gambianus* and *Praomys derooi*, respectively.

			**Rra**	**Rno**	**Mna**	**Mus**	**Cro**	**Cga**	**Pde**	**All small mammals**
Age	Adults	N	133	54	19	88	105	3	4	**406**
		Tox+ (%)	15 (11.3)	4 (7.4)	3 (15.8)	19 (21.6)	26 (24.8)	1 (33.3)	2 (50)	**70 (17.2)**
		Tryp+ (%)	75 (56.4)	16 (29.6)	7 (36 .8)	4 (4.5)	8 (7.6)	2 (66.7)	0	**112 (27.6)**
		Tox+ &Tryp+ (%)	6 (4.51)	1 (1.9)	2 (10.5)	0	3 (2.9)	0	0	**12 (3.0)**
	Juveniles	N	82	12	6	5	7	3	0	**115**
		Tox+ (%)	4 (4.9)	4 (33.3)	0	1 (20)	0	0	-	**9 (7.8)**
		Tryp+ (%)	43 (52.4)	8 (66.7)	3 (50)	1 (20)	1 (14.3)	0	-	**56 (48.7)**
		Tox+ &Tryp+ (%)	2 (2.4)	4 (33.3)	0	1 (20)	0	0	-	**7 (6.1)**
Sex	Males	N	114	27	13	47	40	4	0	**245**
		Tox+ (%)	9 (7.9)	2 (7.4)	0	10 (21.3)	12 (30)	0	-	**33 (13.5)**
		Tryp+ (%)	64 (56.1)	12 (44.4)	5 (38.5)	3 (6.4)	3 (7.5)	1 (25)	-	**88 (35.9)**
		Tox+ &Tryp+ (%)	3 (2.6)	1 (3.7)	0	1 (2.1)	1 (2.5)	0	-	**6 (2.5)**
	Females	N	118	39	14	52	78	3	4	**308**
		Tox+ (%)	14 (11.9)	6 (15.4)	3 (21.4)	10 (19.2)	15 (19.2)	1 (33.3)	2 (50)	**51 (16.6)**
		Tryp+ (%)	64 (54.2)	12 (30.8)	7 (50)	3 (5.8)	6 (7.7)	1 (33.3)	0	**93 (30.2)**
		Tox+ &Tryp+ (%)	7 (5.9)	4 (10.3)	2 (14.3)	0	2 (2.7)	0	0	**15 (4.9)**
Fleas	Yes	N	35	33	4	5	3	0	0	**80**
		Tox+ (%)	3 (8.6)	2 (6.06)	1 (25)	0	1 (33.3)	-	-	**9 (11.3)**
		Tryp+ (%)	26 (74.3)	5 (15.2)	2 (50)	1 (20)	1 (33.3)	-	-	**38 (47.5)**
		Tox+ &Tryp+ (%)	1 (2.9)	1 (3.0)	1 (25)	0	0	-	-	**4 (5)**
	No	N	197	33	23	94	115	7	4	**473**
		Tox+ (%)	20 (10.2)	4 (12.1)	2 (8.7)	20 (21.3)	26 (22.61)	1 (14.3)	2 (50)	**75 (15.9)**
		Tryp+ (%)	102 (51.8)	16 (48.5)	10 (43.5)	5 (5.3)	8 (7.0)	2 (28.6)	0	**143 (30.2)**
		Tox+ &Tryp+ (%)	9 (4.6)	3 (9.1)	1 (4.4)	1 (1.1)	3 (2.61)	0	0	**17 (3.6)**
